# Tobacco sales in pharmacies: a survey of attitudes, knowledge and beliefs of pharmacists employed in student experiential and other worksites in Western New York

**DOI:** 10.1186/1756-0500-5-413

**Published:** 2012-08-06

**Authors:** Danielle M Smith, Andrew J Hyland, Cheryl Rivard, Edward M Bednarczyk, Peter M Brody, James R Marshall

**Affiliations:** 1 Department of Health Behavior, Roswell Park Cancer Institute, Buffalo, NY, USA; 2 School of Pharmacy and Pharmaceutical Sciences, SUNY Buffalo, Buffalo, NY, USA

**Keywords:** Tobacco sales, Pharmacists, Preceptors, Public health policy, Survey research, Pharmacies

## Abstract

**Background:**

Pharmacies are venues in which patients seek out products and professional advice in order to improve overall health. However, many pharmacies in the United States continue to sell tobacco products, which are widely known to cause detrimental health effects. This conflict presents a challenge to pharmacists, who are becoming increasingly more involved in patient health promotion activities. This study sought to assess Western New York (WNY) area pharmacists’ opinions about the sale of tobacco products in pharmacies, and pharmacists’ opinions on their role in patient smoking cessation.

**Methods:**

Participants responded to two parallel surveys; a web-based survey was completed by 148 university-affiliated pharmacist preceptors via a list based sample, and a mail-based survey was completed by the supervising pharmacist in 120 area pharmacies via a list-based sample. The combined response rate for both surveys was 31%. Univariate and bivariate analyses were performed to determine any significant differences between the preceptor and supervising pharmacist survey groups.

**Results:**

Over 75% of respondents support legislation banning the sale of tobacco products in pharmacies. Over 86% of respondents would prefer to work in a pharmacy that does not sell tobacco products. Differences between preceptor and supervising pharmacist groups were observed. Action regarding counseling patients was uncommon among both groups.

**Conclusions:**

Pharmacists support initiatives that increase their role in cessation counseling and initiatives that restrict the sale of tobacco products in pharmacies. These data could have important implications for communities and pharmacy practice.

## Background

Tobacco use is the single largest preventable cause of early death in the U.S. today [1]. Perceptions concerning cigarettes and tobacco use have changed since the 1950’s when tobacco companies used physicians and invoked science in their advertising campaigns [2]. Yet as attitudes towards tobacco have changed, so has the role of the pharmacist. That role once consisted solely of dispensing medications; it now includes counseling patients. This change in role in counseling among pharmacists is now increasingly important with providing smoking cessation counseling, given the change in Nicotine Replacement Therapy (NRT) from being previously being available by prescription, and now being available over-the-counter [3]. This places pharmacists in a unique position to provide tobacco cessation counseling at point-of-sale. Training programs for smoking cessation counseling are increasingly prevalent in schools of pharmacy across the U.S., and more graduates of these schools are being taught the necessary skills to counsel patients on quitting smoking [4]. However, even though they possess knowledge on tobacco cessation counseling, few pharmacists actually engage patients, citing several potential barriers [4-7]. Other research has shown that the majority of pharmacists do not discuss tobacco use with new patients [5].

In other countries, tobacco cessation counseling training for pharmacists have progressed more rapidly, as many nations have a more focused preventative medicine framework within their health care systems. For example, in Finland, the Finnish Current Care Guideline (which outlines smoking cessation responsibilities for all health care professionals) states that pharmacy owners should arrange for tobacco cessation training for their pharmacy staff [8]. Additionally, a 1998 study conducted by researchers in Scotland found that pharmacy staff who underwent training for smoking cessation based on the “stages-of-change” model were more readily able to identify and assist in patients efforts to quit using tobacco than pharmacy staff who did not undergo such formal training [9]. Observed levels of engaging patients for tobacco cessation counseling also appear to be higher in countries outside of the U.S., a likely reflection on smoking cessation training efforts [5-7,10]. For example, a Canadian study found that within their sample of pharmacists, fewer than 50% of pharmacists in the provinces studied intervened with more than half of their smoking patients [10]. This same group found that nearly 44% of current smokers surveyed would be very or somewhat likely to ask a pharmacist for advice on quitting smoking, making pharmacists a somewhat trusted source for solicitation of quit advice in some areas [11]. A 2011 systematic review of public health practices in community pharmacies conducted by a research group in the United Kingdom indicates that sets of public health services, including providing smoking cessation counseling, are now required by community pharmacists in some areas of the U.K. due to the establishment of a new pharmacy contract [12]. This suggests that the role of pharmacists in population-health objectives are more widely acknowledged and advanced in these nations, and training in public health practice in community pharmacies are more heavily emphasized.

 The sale of tobacco products in pharmacies in any locality may send conflicting messages to consumers who come to pharmacies in search of medication or health promotion products. Most U.S. pharmacists oppose the sale of tobacco products in pharmacies [13-16]. Nonetheless, many pharmacies in the United States still sell tobacco products, and polls of pharmacists and pharmacy students show that these groups feel that selling tobacco products in pharmacies violates their personal and professional values [14,17].

Research has found that chain pharmacies are more likely than others to sell tobacco [18]. Pharmacists who work in chain pharmacies also have the least influence on tobacco product sales [18]. A study conducted in 2003, a repeat of a study conducted in the same county in 1976 [19], found that, although the overall percentage of pharmacies selling tobacco products decreased, this change was due mainly to sales decreases in independently owned pharmacies and pharmacies in medical clinics. Chain pharmacies increased in numbers, though there was no change in the percentage of them selling tobacco products [19].

In 1971, the American Pharmacists Association (APhA) House of Delegates released a statement, declaring: “APhA recommends that tobacco products not be sold in pharmacies.” [20,21]. This statement has been periodically renewed to keep smoking cessation a priority [21]. The APhA also recommends that pharmacists “provide services, education, and information on public health issues”, which supports the pharmacist role in providing tobacco cessation services to patients [20]. The American Society of Health-System Pharmacists (ASHP) advocates that pharmacists should become involved in smoking cessation counseling and include patients’ tobacco usage in patient profiles [22]. Although a large percentage of pharmacists are opposed to the sale of tobacco products in pharmacies, and pharmacy organizations have spoken out against this practice, studies still show that, in some areas, more than half of pharmacies sell tobacco products [13,14,16-18]. As students are taught about smoking cessation counseling and the importance of this practice in pharmacies, the contradiction of tobacco sales in pharmacies is becoming more apparent. The American Association of Colleges of Pharmacy (AACP) passed a resolution in 2003 recommending that schools of pharmacy only choose experiential sites for students that do not sell tobacco products [17]. However, student experiential sites are selling tobacco products, and whether these sites are effectively counseling patients on smoking cessation remains important, because pharmacists could play a more decisive role than they do. Additionally, sites that guide students through a university affiliate may provide a differential level of experiential education, as these preceptors are subject to standards and guidelines to which other, non-university affiliated sites may not be subject.

The continued availability of tobacco products within pharmacies in the U.S., including student experiential sites, is due to a host of different factors. These include, but are not limited to: the voluntary nature of businesses in the U.S. adopting recommendations by professional organizations such as the APhA; pharmacies located in “big box” stores like Wal-Mart not having the freedom to choose what products are sold in their workplaces due to corporate business interests [23]; and added incentive for pharmacies to stock tobacco products due to additional revenue provided from displaying tobacco product marketing displays and advertisements [24].

Recently, the United States passed the Family Smoking Prevention & Tobacco Control Act (FSPTCA) of 2009, [25] which gave the FDA and state and local jurisdictions the power to regulate aspects of the sale and marketing of tobacco products. For example, communities can prohibit the sale of tobacco products in pharmacies; this has taken place in Boston and San Francisco [26,27]. While other countries have managed to implement voluntary tobacco sales bans within pharmacies in some areas [10], this is relatively new legislation that gives similar authority to establish such bans within the United States. This legislation could have important implications regarding tobacco sales in pharmacies in the U.S., and aligning the pharmacy as a health promotion resource per APhA recommendations.

### Objectives

The purpose of this study was to establish preliminary data for the Western New York (WNY) area, which included addressing the following research objectives: 1) How do pharmacists feel about the sale of tobacco products in pharmacies?; 2) How do pharmacists view their role regarding patient smoking cessation?; 3) Do thoughts on these issues differ based on the type of work setting in which the pharmacist is employed?; And 4) Do non-university affiliated sites provide on the job training to pharmacy students in outlets where tobacco is sold and where smoking cessation counseling is practiced?

## Methods

The Survey Research and Data Acquisition Resource (SRDAR), within the Department of Health Behavior in the Cancer Prevention Research Program at Roswell Park Cancer Institute, administered two similar surveys to local area pharmacists from October 2010 to December 2010 to address these questions. Sample members were identified through 2 lists provided by the University at Buffalo (UB) School of Pharmacy and Pharmaceutical Sciences. The preceptor sample consisted of a list of contact information for 530 eligible preceptors affiliated with the school. (“Preceptors” are volunteer providers of experiential education within their own worksite that have gained approval from the university, and are subject to adhering to particular guidelines and standards for their worksite and practices as governed by the school.) This web-based survey was sent to all pharmacist preceptor sample members. (Additional file 1) Participants received a survey invitation via e-mail, which included a direct link to the survey online. Reminder e-mails were issued 5 days and 12 days after the initial e-mail was sent; a total of 148 preceptors participated in the survey. The response rate for the web-based preceptor survey was 28%. The supervising pharmacist sample was list-based, and included 345 local area pharmacies for which contact information was available for the supervising pharmacist. This was a mail-based survey sent to all supervising pharmacist sample members in the Western New York Area. (Additional file 2) Potential participants were mailed a survey package via the United States Postal Service containing an advance letter, survey instructions, and the survey instrument. Two weeks after the initial mailing was sent, a reminder letter was sent out to sample members who had not yet responded; a total of 120 pharmacists responded to this survey. The response rate for the mail-based supervising pharmacist survey was 35%. Passive consent was obtained upon receipt of the completed survey, and no compensation was provided. Approval for this research was obtained from Institutional Review Boards at both Roswell Park Cancer Institute and the University at Buffalo.

### Measures

Our team crafted the survey instruments and unique questions to address the specific study aims mentioned previously. As one aim was to collect preliminary data for this project, the questionnaires were not pilot tested prior to fielding. Survey questions in the WNY Pharmacist and UB Pharmacy Preceptors surveys sought to assess the respondent’s professional experience and smoking status, and characteristics of the employing pharmacy. The surveys also included questions about patient interactions surrounding tobacco use, opinions about sales in pharmacies and actions taken to help smokers quit.

### Sale of tobacco products in student experiential sites and other settings

The UB Pharmacy Preceptors survey was administered to pharmacists in student experiential sites. We also asked WNY Pharmacy Survey respondents if their worksite takes student pharmacists for experiential rotations. We asked all participants, “Which of the following best describes your current work setting?”, and used this information to group respondents into 3 distinct categories for analysis. The first category consisted of those employed in “chain” and other retail settings; defined as those who work in chain drug stores, pharmacies in large grocery stores, and those at other retail pharmacies located in big-box retailers. The second group included those who indicated they work in independently-owned community pharmacies. The third group consisted of those employed in non-retail settings, such as clinically-affiliated sites in hospitals or physician offices.

All participants were asked if their place of employment sold cigarettes and non-prescription nicotine replacement therapy (NRT). WNY Pharmacist Survey respondents were also asked whether their pharmacy or store sells tobacco products other than cigarettes, and whether the site sells alcohol.

### Role of the pharmacist in tobacco cessation counseling

1. Actions taken regarding patient smoking:

To determine the types of action taken by pharmacists presented with a patient who smokes, we asked all respondents questions regarding the requirement and frequency with which tobacco use is documented in patient records within worksites. We also asked questions to determine actions regarding inquiring about tobacco use when patients come in to obtain both prescription and over the counter (OTC) medications. The New York State Department of Health, in collaboration with Roswell Park Cancer Institute, supports a free, telephone-based tobacco cessation service; The New York State Smokers’ Quit line. Callers can receive printed tobacco-cessation guidelines, advice and counseling, and free start-up packages of nicotine-replacement treatment. Health care providers are encouraged to refer those who smoke to the Quit line to get assistance with smoking cessation, therefore, we also asked respondents, “How often do you refer patients who use tobacco to the (NY State) Quit line or other cessation service?” WNY Pharmacist Survey respondents were also asked, “How often do you provide smoking cessation counseling?”

2. Beliefs about the role of the pharmacist in patient smoking cessation:

Respondents for both surveys were asked how much they agree or disagree with statements pertaining to all pharmacists, most pharmacists, and themselves as pharmacists taking an active role in helping people quit using tobacco.

3. Reported barriers to providing tobacco cessation counseling:

All participants were asked whether the following 8 factors posed a barrier to providing tobacco cessation counseling to patients (answer choices being “Not a barrier”, “Somewhat or occasionally a barrier”, or “Definitely or often a barrier” for each issue presented): 1) “Lack of time to provide counseling/overburdened with other duties”; 2) “Pharmacy is not adequately staffed”; 3) “Don’t believe counseling is effective”; 4) “Lack of support from upper management”; 5) “Uncomfortable initiating conversation about a patient’s tobacco use”; 6) “Lack of training for cessation counseling”; 7) “Patient’s lack of time for counseling/in a hurry”; and, 8) “Patients feel it is intrusive/not a pharmacist’s business”. WNY Pharmacist Survey participants were also asked about an additional issue: “Lack of reimbursement for smoking cessation counseling”.

### Support for policies banning the sale of tobacco products in pharmacies

All respondents were asked if it is inappropriate to sell tobacco products in community chain drug stores, community independent drug stores, and grocery stores and wholesale stores with pharmacies in them, respectively. We also asked whether respondents felt it was important to provide products sought about by consumers (including tobacco), and their preference for working in a venue that does not sell tobacco. UB Pharmacy Preceptors Survey participants were also asked their opinion on the additional statement, “It is inappropriate for businesses with pharmacies in them to display ads and promotions for tobacco.” We asked all participants directly whether they would strongly support, support, oppose, or strongly oppose legislation banning the sale of tobacco in pharmacies.

### Other covariates

Demographic covariates included gender (male vs. female), smoking status (current, former, or never smoker), level of education (B.S., M.S., Pharm D., Other Doctorate, or Residency Training), School Location (at UB, School in NYS, School outside of NYS, School outside of U.S.), Years licensed (1–5 years, 6–15 years, 16–25 years, 26+ years), and having received formal tobacco cessation counseling (yes vs. no). Respondents were classified as smokers if they indicated they had smoked at least 100 cigarettes in their entire life, and smoke either “every day” or “some days”.

### Statistical analysis

Descriptive statistics were assessed for all measures by means of SPSS v. 14.0. All measures were examined according to survey version (UB Pharmacy Preceptors Survey vs. WNY Pharmacist Survey) and type of work setting (employed at a chain retailer/other retail setting, independently owned pharmacy, or non-retail setting, which we defined as clinically-affiliated sites, such as those located within hospitals or physician offices.), to assess for any significant differences in practices between the preceptor and supervising pharmacist groups. Frequencies, cross tabulations, and chi-square tests of independence were used to test for the significance of differences between both survey group and among the retail settings in which the respondents were employed. Due to their small number in the WNY Pharmacist Survey, those employed in non-retail settings were excluded from this report. To maximize the comparability of our results, we also examined the subset of sample members employed in retail settings separately for any significant differences between the preceptor and supervising pharmacist groups.

## Results

### Respondent demographic and work site characteristics

Respondents in the two groups are relatively similar in terms of gender, smoking status, years licensed and training in tobacco cessation. (Additional file 3: Table 1) Current smoking is uncommon; a sizeable majority of respondents are never smokers. Only about forty percent of respondents—whether pharmacy preceptors or supervising pharmacists—have had formal training in tobacco cessation counseling, with significantly higher numbers of preceptor respondents employed in chain settings having received such training (57%). Preceptors are less likely than supervising pharmacists to work in a chain retail setting, or in an independently owned pharmacy; they are much more likely to work in a non-retail setting.

Although preceptors are less likely than supervising pharmacists to work in a retail or independent pharmacy, an appreciable number of preceptors work in these settings. (Additional file 4: Table 2) The data confirm that smoking is uncommon among both pharmacy preceptors and supervising pharmacists. Preceptors in retail settings are more likely than preceptors in independent or non-retail pharmacies, and more likely than supervising pharmacists, to have formal tobacco counseling training. All of the preceptor pharmacists accept student pharmacists for training, but well over half of supervising pharmacists are in settings that accept student trainees. Tobacco is sold in the great majority of chain retail outlets of both preceptors and supervising pharmacists; it is generally not sold in independently owned pharmacies. Preceptors were not asked about the sale of other tobacco products; most of the supervising pharmacists are in outlets in which other tobacco products are sold. Non-prescription nicotine replacement is sold in almost all the retail pharmacies in which respondents are employed. Although pharmacies in New York are encouraged to carry and display promotional materials for the Quit line, only about half of the pharmacies of Western New York respondents display these materials.

### Pharmacist activities regarding patient tobacco use and provision of cessation services and information

Virtually no respondents report being required to document tobacco use of patients, or to enter such information into patient records. (Additional file 5: Table 3) Although preceptor pharmacists are more likely than supervising pharmacists to document patient tobacco use, even they are extremely unlikely to do so. Data also indicates that respondents do not generally ask patients counseled for over-the-counter or prescriptions medications about tobacco use; the percentages of respondents who report “always/ usually” asking about tobacco are small. Preceptors are more likely than supervising pharmacists to inquire about patient tobacco use, a finding which was significantly different between those preceptors and supervising pharmacists employed in chain retail settings, but over three fourths report either that they “sometimes” or “rarely/never” ask about tobacco use. Respondent pharmacists usually do not refer patients heavily to the New York State Quit line: only 22 percent of preceptors indicate that they “always/usually” refer tobacco-using patients to the Quit line, and only 11 percent of supervising pharmacists do.

### Reported barriers to providing smoking cessation counseling

Table 4 (Additional file 6: Table 4) indicates that there are only modest statistically non-significant differences between preceptors and supervising pharmacists in the extent to which time demands are described as barriers to counseling; 21 percent of preceptors, and 27 percent of supervising pharmacists report that time demands are definitely or often a barrier to providing cessation counseling.

The results displayed in Table 4 (Additional file 6: Table 4) also indicate that respondents are unsure about the importance of the patient’s lack of time to be counseled: nearly one-third of respondents identified patient lack of time as definitely or often a barrier to counseling about cessation. On the other hand, pharmacists are likely not to see patient objections over pharmacist intrusiveness as a major barrier to cessation counseling; 42 percent of preceptors, and 27 percent of supervising pharmacists describe patient concerns over intrusiveness as not a barrier to counseling, a non-significant difference. Substantial proportions of responders are likely to be unsure about patient feelings of intrusiveness; 42 percent of preceptors and 53 percent of supervising pharmacists agree that patient feelings of intrusiveness are a possible or occasional barrier to counseling. Very few respondents—fewer than 20 percent in either group-describe patient concerns as a definite barrier to cessation counseling. Over 50% of supervising pharmacists indicated that “Lack of Reimbursement for smoking cessation counseling” was not a barrier to providing this service.

### Opinions about the sale of tobacco products in pharmacies

It can be seen that there is some divergence of opinion on the inappropriateness of tobacco sales in pharmacies. (Additional file 7: Table 5) Generally, between 70 and 80 percent of pharmacists regard tobacco sales in pharmacies as inappropriate. They are less likely to see tobacco sales as inappropriate in grocery stores and other retail outlets with pharmacies in them. In even these, however, at least 50 percent of pharmacists describe tobacco sales as inappropriate. Just over 86% of all survey respondents indicated that they would prefer to work in a venue that does not sell tobacco. With respect to pharmacist opinion on whether tobacco sales in pharmacies should be legally banned, our data show that just over 75% of respondents support such legislation. When restricting the analysis to just those employed in retail settings, statistically significant differences in opinions were observed between preceptors and supervising pharmacists employed in those environments.

## Discussion

This report describes the attitudes of a potentially important leader in the struggle against what is likely the most devastating public health threat of the 20^th^ and now the 21^st^ century: cigarette smoking. We sampled from two important sectors of the pharmacy practice community of Western New York: preceptors of the University at Buffalo School of Pharmacy, and supervising pharmacists at Western New York pharmacies.

The results indicated that preceptors, as members of a recognized and highly respected academic community, tended to have more education, were more likely to have completed a residency, and were more likely to engage in tobacco cessation counseling activities than supervising pharmacists. The pharmacies where preceptors and supervising pharmacists work are about equally likely to sell tobacco products and nicotine replacement treatments. In retail settings, preceptors were more likely than supervising pharmacists to have received formal tobacco cessation counseling, although the difference between these two groups is slight. These are differences that may influence the quality of experiential education that pharmacy students receive, particularly with respect to applied training for the provision of smoking cessation counseling to patients.

A striking component of these findings is that preceptors are more likely than supervising pharmacists to be familiar with patient tobacco use, and to take rudimentary steps to advise regarding tobacco use. Even with the presence of confounding with respect to work setting and preceptor designation, measures of association still point toward preceptors more actively engaging in smoking cessation activities than pharmacists not enrolled in the preceptor program. Preceptors are more likely than supervising pharmacists to ask prescription patients about tobacco use and to refer tobacco-using patients to the New York State Quit line. Therefore, students receiving training by those in the university-affiliated preceptor group employed in retail settings are likely being exposed to tobacco cessation counseling practices through their mentors at a slightly higher level than those who are not receiving training with university affiliates. Nonetheless, the percentages of even preceptors who report that they always or usually take such actions are very low: below one fourth. Half to three fourths of even preceptors report that they rarely or never record tobacco use, ask about tobacco use, or refer patients to the New York State Smoker Quit line. Other studies have shown similar results [3-6,10]. Ninety percent of the pharmacies of preceptors, even at this internationally recognized school of pharmacy, are not required to document tobacco use.

These data offer no easy answers as to why pharmacists do not take a more active role in addressing patient tobacco use. Among retail preceptors and supervising pharmacists, few are likely to report that lack of time, inadequate staffing, lack of upper management support, discomfort over initiating a conversation about tobacco use or lack of training is definitively or often a barrier to providing tobacco cessation counseling. About a third of pharmacists report concern over patient time, and over the possibility that patients might feel like pharmacist advice regarding tobacco use might be intrusive. In fact, one interesting finding from this study was that over 50% of supervising pharmacists reported that lack of reimbursement for smoking cessation counseling was not a barrier to providing such services, which is consistent with what other studies have shown in the U.S. and in other nations [12]. Our discomforting finding is that local area pharmacists do not take advantage of the opportunity to educate and counsel patients regarding tobacco use. We need to better understand why such action is not undertaken. Would more cessation training help? Should such training be mandatory? Although our current data do not address such questions, these are questions that warrant further research. For example, in other areas, research has suggested that economic incentives for pharmacists to provide such counseling may provide additional motivation for the provision of such services to patients, as would the provision of an appropriate, private setting in which counseling sessions could be conducted [12]. Responses from participants in our study are also consistent with that of other studies with regard to barriers to providing cessation counseling involving patient-pharmacist interaction. Other research studies have mentioned that of those patients who have received public health services (such as tobacco cessation counseling) in community pharmacy settings, the response to such services by patients has been largely positive [12]. Eades et al. mention that additional training for pharmacists to engage in such public health practices in their work sites may boost confidence in the provision of such services, and this confidence may yield higher levels of counseling practices. Additionally, this team mentions that these training sessions may include a review of research indicating the positive consumer experiences of those who have been recipients of public health services may be helpful in motivating pharmacists to provide such services such as smoking cessation counseling [12].

In spite of their relative inaction, both preceptor and supervising pharmacists regard tobacco sales in pharmacies as inappropriate. Pharmacists as health professionals are fully aware of the myriad pathogenic effects of tobacco, and they recognize the conflict between their roles as health professionals and as merchants dispensing what is clearly a major preventable pathogen. Their position is in this vein consistent with the position of the American Pharmaceutical Association, that pharmacies should not be selling tobacco products. Despite this point, our data show that 80% of preceptors and 85% of supervising pharmacists that work in chain retail pharmacies indicated that tobacco products were sold in their worksites. As mentioned, other studies have shown that pharmacists employed in chain retail outlets have little influence on the products that are sold within their work sites [18]. However, nearly all pharmacists employed in chain retail and independently-owned pharmacies included in our study reported that their site sold non-prescription NRT. As mentioned by other research conducted outside of the United States [10,11], this can serve as an important tool for pharmacists to encourage their own involvement in patient smoking cessation counseling.

Data collected in New York indicate that tobacco sales are a decreasing source of pharmacy income [28]. It may be the modesty of income, but is more likely the inconsistency of the pharmacist’s ethical code regarding tobacco merchandising that overwhelming majorities of both preceptor and supervising pharmacists voice agreement with the establishment of laws banning pharmacy tobacco sales. Other studies have shown this [13-16].

### Limitations

The current study is subject to some limitations. The combined response rate for both surveys (web and mail-based) was 31% (28% for preceptor respondents, 35% for supervising pharmacist respondents.) A factor for non-response may be that no incentive was offered [29]. This response rate lead to a final sample size of 268 participants. Although we did observe statistically significant differences throughout our analyses, some cell sizes were small when performing stratified analyses. Sample size was also a limiting factor in the decision to perform multivariate analyses. The supervising pharmacist survey was mailed to organizational addresses, where other workplace duties may have discouraged respondent participation. The preceptor survey was sent out via e-mail, which could have presented barriers to completion in worksites where computer access was needed for other workplace duties. The differences in the response rates between the preceptor and supervising pharmacist survey may potentially be reflective of these challenges with each survey administration method. Although we do not have information regarding the demographic characteristics of non-responders for this survey, characteristics of our final sample members are relatively similar to other studies conducted amongst pharmacists in the U.S. regarding similar subject matter [13,15,16]. This survey was conducted in a single geographic location, so these results may not pertain to pharmacists in other regions, and this should be considered when interpreting these findings. Yet despite regional limits, these results are consistent with those of other studies [13-18]. Also, it became clear during analysis that preceptor status and work setting are confounded due to the placement of preceptors in particular work settings. We attempted to maximize comparability by performing restricted analysis on the subset of sample members that were employed in retail settings; many of the conclusions drawn from the total sample remained unchanged. Despite this challenge, this report provides a basis through which these issues can be further examined. Many of our findings (such as availability of tobacco in chain pharmacies) are consistent with what other studies have shown [16].

## Conclusions

The New York State Assembly and Senate have considered bills banning tobacco sales in pharmacies, although those bills have not progressed very far. Coupling this information with the enactment of the FSPTCA of 2009 [25], which gives state and local communities the authority to regulate aspects of the sale and marketing of tobacco products, the data in this paper may help inform such policy debates regarding the sale of tobacco products in pharmacies in the WNY area, and other parts of the U.S. In other areas outside of the United States, bans on the sale of tobacco products in pharmacies have already been implemented [10]. Surveys conducted amongst pharmacists in these locations show that pharmacists generally yield more positive attitudes toward smoking cessation counseling practices compared with areas that do not have sales bans [10]. If bans were to be implemented in other areas in the U.S, these may have an impact on smoking cessation counseling practices. While this Canadian study noted that sales bans appeared to have had little effect on intervention levels among pharmacists [10], circumstances faced by pharmacists practicing in the U.S. may yield other results. Regardless of the impact on practice, tobacco sales in pharmacies continue to be inconsistent with the role of the pharmacist as a health care provider, and these data could go on to inform policy that will help align their role within public health practice.

The information presented in this report may also serve as a basis for those in other institutions to examine the environmental characteristics and behaviors by pharmacists that take on student trainees to determine what student trainees are exposed to in these sites. Other organizations may also wish to examine pharmacists’ opinions about the sale of tobacco in their workplaces in their own communities, which may give data to inform community policy debates in other geographic areas.

## Competing interests

The authors declare that they have no competing interests.

## Authors’ contributions

EB and PB developed the concept for this study and assisted in its design and the development of the survey instruments. CR lead the development of the survey instruments, obtained the necessary IRB approval from boards at both Roswell Park Cancer Institute and the University at Buffalo, coordinated data collection efforts, and assisted with drafting the manuscript. DS performed the statistical analysis and coordinated the drafting of the manuscript. AH participated in the design of the study, contributed to the drafting of the manuscript, and oversaw the project. JM contributed to the design of the study, drafting of the manuscript, and contributed to the data analysis. All authors reviewed and approved the final draft.

## Supplementary Material

Additional file 1 Survey Instrument; administered via web to University at Buffalo Pharmacy Preceptors, 2010. Click here for file

Additional file 2 Survey Instrument, administered via mail to Western New York Area Supervising Pharmacists, 2010. Click here for file

Additional file 3 **Table 1.** Characteristics of survey participants and worksites (n=268). Click here for file

Additional file 4 **Table 2.** Characteristics of pharmacies employing survey respondents (n=268). Click here for file

Additional file 5 **Table 3.** Actions taken by pharmacists regarding patient smoking and reported barriers to providing smoking cessation counseling (n=186). Click here for file

Additional file 6 **Table 4.** Reported barriers to providing tobacco cessation counseling (n=183). Click here for file

Additional file 7 **Table 5.** Pharmacists’ beliefs about the sale of tobacco products in pharmacies (n=268). Click here for file
