# Supplementary material for: Tobacco sales in pharmacies: a survey of attitudes, knowledge and beliefs of pharmacists employed in student experiential and other worksites in Western New York
Source: BMC Res Notes. 2012 Aug 6;5:413. doi: 10.1186/1756-0500-5-413 (PMC3492148; doi:10.1186/1756-0500-5-413)
Supplement: Additional file 1 — Survey Instrument; administered via web to University at Buffalo Pharmacy Preceptors, 2010. [file 1756-0500-5-413-S1.doc]

# Section 1. Professional Experience

1. Are you a:

1. Pharmacist

2. Pharmacy Technician

2. What college or graduate degree(s) have you attained? (Circle **all** that apply)

1. B.S. or B.A.

2. M.S. or M.A. or equivalent

3. Pharm.D.

4. Residency training

5. Doctorate other than Pharm.D. (e.g., Ph.D.)

6. No degree

3. Where was the majority of your schooling done? (circle one)

1. At UB

2. At a school in New York (not UB)

3. At a school outside of New York.

4. At a school outside the U.S.

4. How many years have you been licensed as a pharmacist or pharmacy technician in the State of
New York? Please round to the nearest year. _________ years

# 5. What is your gender?

# 1. Male

# 2. Female

# Section 2. Work Environment: Community and Outpatient Pharmacies

6. Which of the following best describes your **current** work setting(s)?

1. Community, Chain drug store (i.e. Walgreen’s, Rite Aid, etc.)

2. Community, Independently owned drug store

3. Community, Grocery store/ market (i.e. Tops, Wegman’s, etc.)

4. Community, Wholesale store (i.e. Costco, Sam’s Club, etc.)

5. Community, Clinically affiliated site (i.e. physician’s office)

6. Non-community setting (continue to Section 6)

7. Other community pharmacy-related setting: ____________________________

7. How many full-time pharmacists (35 hours or more per week) work at your **primary** work setting? 1-2 3–4 5 or more

8. How many part-time pharmacists (less than 35 hours per week)
work at your **primary** work setting? 1-2 3–4 5 or more

9. About how many prescriptions does your pharmacy fill in an average weekday? _________ Rx/day

10. Does the place where you are primarily employed sell tobacco products?

1. Yes

2. No

11. Does your work site sell **nonprescription** nicotine patches or gum?

1. Yes

2. No

12. Does your place of employment display posters or other promotional materials for the New York State Quitline or other tobacco cessation services?

1. Yes

2. No

13. Are your pharmacy staff required to document tobacco use in a patients’ record or profile at intake?

1. Yes

2. No

# Section 4. Patient Interactions Surrounding Tobacco Use

14. Do you interact regularly with patients? (Circle one number)

1. Yes

2. No ⮊ IF NO, **go to Section 6.**

15. Never Rarely Sometimes Usually Always

a. How often do you ask persons with **prescriptions** (for any
 type of medication) whether they use tobacco? 0 1 2 3 4

b. How often do you ask people whom
 you counsel for OTC medication whether they use tobacco? 0 1 2 3 4

c. How often do you document tobacco use in a patients’ profile
 or record? 0 1 2 3 4

d. How often do you refer patients who use tobacco to the
 state’s Quitline or other cessation service? 0 1 2 3 4

**Section 5. Barriers to Providing Counseling**

16. For you, how much of a barrier to providing tobacco cessation counseling are each of the following?

|  | Not a barrier | Somewhat or occasionally a barrier | Definitely or often a barrier |
| --- | --- | --- | --- |
| a. Lack of time to provide counseling/overburdened with other duties | 1 | 2 | 3 |
| b. Pharmacy is not adequately staffed | 1 | 2 | 3 |
| c. Don’t believe counseling is effective | 1 | 2 | 3 |
| d. Lack of support from upper management | 1 | 2 | 3 |
| e. Uncomfortable initiating conversation about a patient’s tobacco use | 1 | 2 | 3 |
| f. Lack of training for cessation counseling | 1 | 2 | 3 |
| g. Patient’s lack time for counseling/are in a hurry | 1 | 2 | 3 |
| h. Patients feel it is intrusive/not a pharmacist’s business | 1 | 2 | 3 |
|  |  |  |  |

What else might get in the way of your providing cessation counseling? _______________________________

__________________________________________________________________________________________

# Section 6. Your Opinions

17. Pharmacists **should** take an active role in helping people quit using tobacco.

1. Strongly agree

2. Agree
 3. Disagree

4. Strongly disagree

18. Most pharmacists **currently** take an active role in helping people quit using tobacco.

1. Strongly agree

2. Agree
 3. Disagree

4. Strongly disagree

19. What is your opinion regarding the following statements?

Strongly Strongly

Agree Agree Disagree Disagree

1. It is inappropriate for community chain drug stores to sell tobacco products. . . . . . . . 1 2 3 4

It is inappropriate for community independent drug stores to sell tobacco products. . . . . . . . 1 2 3 4

1. It is inappropriate for grocery stores and wholesale stores with pharmacies in them to sell tobacco products. . . . . . . . 1 2 3 4
2. It is inappropriate for businesses with pharmacies to display ads and promotions for tobacco. 1 2 3 4
3. It is important to provide the products that people want, even if it includes tobacco products. 1 2 3 4
4. All else being equal, I would prefer to work in a
   pharmacy that did not sell tobacco products. 1 2 3 4

20. Several governments have passed legislation banning the sale of tobacco in pharmacies. Do you support or oppose this type of regulation?

1. Strongly support

2. Support

3. Oppose

4. Strongly oppose

**Section 7. Demographics**

21. Have you smoked at least 100 cigarettes in your entire life? (100 cigarettes = 5 packs)

1. Yes – ask 22.

2. No – skip to 25.

3. Don’t know/ Not sure - ask 22.

22. Do you now smoke cigarettes every day, some days, or not at all?

1. Every day – skip to 25.

2. Some days – skip to 25.

3. Not at all

1. If you no longer smoke cigarettes, how many years did you smoke?
   1. <5
   2. 6-10
   3. 11+
2. If you no longer smoke cigarettes, approximately how many years ago did you quit?

25. Have you had formal training for tobacco cessation counseling? (Circle one number)

1. Yes

2. No

26. Does your pharmacy sell tobacco products?

1. Yes

2. No

27. What would be the most notable impact (both positive and negative) on your place of employment if tobacco products were no longer sold? (if your work site does not sell tobacco, please leave blank)
 _______________________________________________________________________________________

 _______________________________________________________________________________________

28. How would your relationship with people at your site (both tobacco using and non-tobacco using patrons) be affected if your work site no longer sold tobacco products?

_______________________________________________________________________________________

 _______________________________________________________________________________________

Thank you for taking the time to complete this survey. Please fax to xxx-xxx-xxxx

If you prefer, you can mail your survey to:
Gilmore Research Group

2324 Eastlake Ave. East, Suite 300
Seattle, WA 98102
