# Supplementary material for: Tobacco sales in pharmacies: a survey of attitudes, knowledge and beliefs of pharmacists employed in student experiential and other worksites in Western New York
Source: BMC Res Notes. 2012 Aug 6;5:413. doi: 10.1186/1756-0500-5-413 (PMC3492148; doi:10.1186/1756-0500-5-413)
Supplement: Additional file 2 — Survey Instrument, administered via mail to Western New York Area Supervising Pharmacists, 2010. [file 1756-0500-5-413-S2.doc]

Instructions: Please use a blue or black pen or #2 pencil to fill in completely

the circle that goes with your answer choice.

# Section 1. Professional Experience

1. What training level have you completed? (Mark **all** that apply)

O B.S.

O M.S. or M.A. or equivalent

O Pharm.D.

O Residency training

O Fellowship

O Doctorate other than Pharm.D. (e.g., Ph.D.)

2. Where was the majority of your schooling done? (Choose one)

O At UB

O At a school in New York

(not UB)

O At a school outside of New York

O At a school outside the U.S.

3. How many years have you been licensed as a pharmacist in the State of New York? Please round to the nearest year.

_________ years

4. What is your gender?

O Male O Female

5. Does your pharmacy accept student pharmacists for experiential rotations (IPPE, APPE, etc) from the University at Buffalo or other Schools of Pharmacy?

O Yes O No

6. If yes, how many students would you estimate your pharmacy precepts per year?

IPPE _____ APPE _____

7. Have you had formal training for tobacco cessation counseling?

O Yes O No

# Section 2. Work Environment

8. Which of the following best describes your **current** work setting? (Choose one)

O Community, Chain drug store

(i.e. Walgreen’s, Rite Aid, etc.)

O Community, Independently

owned drug store

O Community, Grocery store

(Tops, Wegmans, etc)

O Community, general retail store

(Wal-Mart, K-Mart, etc.)

O Other community pharmacy-

related setting: __________ _______________________

9. About how many prescriptions does your pharmacy fill in an average weekday?

_________ Rx/day

1. Does the sale of grocery items make up a substantial portion of your business?

O Yes O No

1. Does your pharmacy/store sell alcohol or alcohol related products?

O Yes O No

1. Does your pharmacy/store sell cigarettes?

O Yes O No

1. Does your pharmacy/store sell other tobacco products?

O Yes O No

1. Does your pharmacy/store

receive incentives from tobacco companies to carry/stock their products?

O Yes O No O Don’t

know

1. If cigarettes or tobacco products are sold in your store, where are they located?

O In the pharmacy

O Near the pharmacy

O In a separate location, not near

the pharmacy

# Section 3. Patient Interactions Surrounding Tobacco Use

16. Does your pharmacy sell **nonprescription** nicotine patches or gum?

O Yes O No

17. Does your pharmacy display posters or other promotional materials for the New York State Smokers’ Quitline or other tobacco cessation services?

O Yes O No

18. Is your pharmacy staff required to document tobacco use in a patient’s record or profile at intake?

O Yes O No

19. Never Rarely Sometimes Usually Always

a. How often do you ask persons with **prescriptions** (for any

type of medication) whether they use tobacco? O O O O O

b. How often do you ask people whom you counsel for OTC

medication whether they use tobacco? O O O O O

c. How often do you document tobacco use in a patient’s

profile or record? O O O O O

d. How often do you refer patients who use tobacco to the

state’s Quitline or other cessation service? O O O O O

e. How often do you provide smoking cessation counseling? O O O O O

Section 4. Barriers to Providing Counseling

20. For you, how much of a barrier to providing tobacco cessation counseling are each of the following?

Somewhat or Definitely occasionally or often

Not a barrier a barrier a barrier

a. Lack of time to provide counseling/ overburdened with

other duties O O O

b. Pharmacy is not adequately staffed O O O

c. Don’t believe counseling is effective O O O

d. Lack of support from upper management O O O

e. Uncomfortable initiating conversation about a

patient’s tobacco use O O O

f. Lack of training for cessation counseling O O O

g. Patient’s lack time for counseling/are in a hurry O O O

h. Patients feel it is intrusive/not a pharmacist’s

business O O O

i. Lack of reimbursement for smoking cessation

counseling O O O

J. Other (for other: Describe) _____________________________ O O O

# Section 5. Your Opinions

21. Pharmacists **should** take an active role in helping people quit using tobacco.

O Strongly agree O Agree O Disagree O Strongly disagree

22. I **currently** take an active role in helping people quit using tobacco.

O Strongly agree O Agree O Disagree O Strongly disagree

Strongly Strongly

23. **What is your opinion regarding the following statements?** Agree Agree Disagree Disagree

a. It is inappropriate for community chain drug stores to sell O O O O

tobacco products

b. It is inappropriate for community independent drug stores to O O O O

sell tobacco products

c. It is inappropriate for grocery stores and wholesale O O O O

stores with pharmacies in them to sell tobacco products

d. It is important to provide the products that people want, O O O O

even if it includes tobacco products.

e. All else being equal, I would prefer to work in a O O O O

pharmacy that did not sell tobacco products.

24. The APhA recently passed a broad resolution opposing the sale of tobacco in pharmacies.

Do you support these positions?

O I am not familiar O Strongly support O Support O Oppose O Strongly oppose

with this resolution

Section 6. Personal Tobacco Use History

25. Have you smoked at least 100 cigarettes in your entire life? (100 cigarettes = 5 packs)

O Yes O No O Don’t know/ Not sure

26. Do you now smoke cigarettes every day, some days, or not at all?

O Every day O Some days O Not at all

1. If you no longer smoke cigarettes, how many years did you smoke?

O never smoked

O <5 years

O 6-10 years

O 11+ years

1. If you no longer smoke cigarettes, approximately how long ago did you quit?

_________ Days (or) _________ Months (or) _________ Years

**Thank you for completing the survey!**
