# Supplementary material for: Tobacco sales in pharmacies: a survey of attitudes, knowledge and beliefs of pharmacists employed in student experiential and other worksites in Western New York
Source: BMC Res Notes. 2012 Aug 6;5:413. doi: 10.1186/1756-0500-5-413 (PMC3492148; doi:10.1186/1756-0500-5-413)
Supplement: Additional file 3 — Table 1. Characteristics of survey participants and worksites (n=268). [file 1756-0500-5-413-S3.docx]

| Table 1: Characteristics of survey participants and worksites^a^ (n=268) | | | |  |  |  |
| --- | --- | --- | --- | --- | --- | --- |
|  |  |  |  |  |  |  |
|  |  | **UB Pharmacy Preceptors Survey** |  | **WNY Pharmacist Survey** |  | **Total** |
|  | **Participant Characteristics^a^** | (% (n=148)) |  | (% (n=120)) |  | (% (n=268)) |
| **Gender** | Male | 52 |  | 63 |  | 57 |
|  | Female | 48 |  | 37 |  | 43 |
| **Smoking Status** | Current Smoker | 3 |  | 2 |  | 2 |
|  | Former Smoker | 18 |  | 20 |  | 19 |
|  | Never Smoker | 79 |  | 79 |  | 79 |
| **Education** | B.S. | 37 |  | 66 |  | 50 |
|  | M.S. | 2 |  | 2 |  | 2 |
|  | Pharm D. | 33 |  | 31 |  | 32 |
|  | Other Doctorate | 3 |  | 2 |  | 3 |
|  | Residency Training | 24 |  | 0 |  | 13 |
| **School Location** | UB | 54 |  | 72 |  | 62 |
|  | School in NYS | 24 |  | 11 |  | 18 |
|  | School Outside NYS | 21 |  | 17 |  | 19 |
|  | School Outside U.S. | 1 |  | 0 |  | 1 |
| **Years Licensed** | 1-5 Years | 25 |  | 19 |  | 22 |
|  | 6-15 Years | 29 |  | 23 |  | 26 |
|  | 16-25 Years | 19 |  | 28 |  | 23 |
|  | 26+ Years | 26 |  | 31 |  | 29 |
| **Tobacco Cessation** | Had formal counseling training | 39 |  | 37 |  | 38 |
|  | No formal counseling training | 61 |  | 63 |  | 62 |
| **Work Setting** | Chain Retailer/Other Retail Setting | 27 |  | 68 |  | 46 |
|  | Independently Owned Pharmacy | 10 |  | 29 |  | 19 |
|  | Non-Retail Setting | 62 |  | 3 |  | 35 |
| ^a^ Underlined values denote statistically significant differences (chi-square test comparing survey versions (p-value<0.05)) | | | | | | |
