# Supplementary material for: Tobacco sales in pharmacies: a survey of attitudes, knowledge and beliefs of pharmacists employed in student experiential and other worksites in Western New York
Source: BMC Res Notes. 2012 Aug 6;5:413. doi: 10.1186/1756-0500-5-413 (PMC3492148; doi:10.1186/1756-0500-5-413)
Supplement: Additional file 4 — Table 2. Characteristics of pharmacies employing survey respondents (n=268). [file 1756-0500-5-413-S4.docx]

| Table 2: Characteristics of pharmacies employing survey respondents ^a^ (n=262) | | | | | |  |  |  |
| --- | --- | --- | --- | --- | --- | --- | --- | --- |
|  | |  |  |  |  |  |  |  |
|  | |  | **UB Pharmacy Preceptors** | | |  | **WNY Pharmacists** | |
|  | |  | **Chain Retailer/Other Retail Setting** | **Independently Owned Pharmacy** | **Non-Retail Setting** |  | **Chain Retailer/Other Retail Setting** | **Independently Owned Pharmacy** |
|  | |  | (n=40) | (n=15) | (n=91) |  | (n=81) | (n=35) |
| **Sell Cigarettes %** | |  | 80 | 0 | 1 |  | 85 | 6 |
| **Sell Other Tobacco Products %^b^** | |  | n/a | n/a | n/a |  | 82 | 6 |
| **Smokers %** | |  | 3 | 14 | 1 |  | 1 | 3 |
| **Formal tobacco cessation counseling training %** | | | 57 | 36 | 32 |  | 42 | 24 |
| **Take On Student Pharmacists %^b^** | | | n/a | n/a | n/a |  | 67 | 56 |
| **Sell Non-Prescription NRT %** | | | 100 | 93 | 39 |  | 100 | 94 |
| **Sell Alcohol %^b^** |  | | n/a | n/a | n/a |  | 94 | 3 |
| **Displaying promotional materials for Quitline %** | | | 40 | 50 | 48 |  | 48 | 52 |
| ^a^ Underlined values note significant association between pharmacy characteristic and type of worksite (p<0.05)  ^b^ These questions were included on the WNY Pharmacist questionnaire only. | | | | | | | |  |
