# Supplementary material for: Tobacco sales in pharmacies: a survey of attitudes, knowledge and beliefs of pharmacists employed in student experiential and other worksites in Western New York
Source: BMC Res Notes. 2012 Aug 6;5:413. doi: 10.1186/1756-0500-5-413 (PMC3492148; doi:10.1186/1756-0500-5-413)
Supplement: Additional file 5 — Table 3. Actions taken by pharmacists regarding patient smoking and reported barriers to providing smoking cessation counseling (n=186). [file 1756-0500-5-413-S5.docx]

| Table 3: Actions taken by pharmacists regarding patient smoking and reported barriers to providing smoking cessation counseling^a^  (n=186) | | | | | | | |  | |
| --- | --- | --- | --- | --- | --- | --- | --- | --- | --- |
|  |  |  |  | **UB Pharmacy Preceptors (Retail Only)** | | **WNY Pharmacists (Retail Only)** | | |  |
|  |  | **Total UB Pharmacy Preceptors^b^** | **Total WNY Pharmacists** | **Chain Retailer/Other Retail Setting** | **Independently Owned Pharmacy** | **Chain Retailer/Other Retail Setting** | **Independently Owned Pharmacy** | |  |
|  |  | (n=68) | (n=118) | (n=40) | (n=15) | (n=81) | (n=35) | |  |
| % required to document tobacco use | | 10 | 2 | 5 | 7 | 3 | 0 | |  |
| How often do you document tobacco use in a patients record? (%) | |  |  |  |  |  |  | |  |
| % always/usually | | 16 | 2 | 6 | 7 | 1 | 3 | |  |
| % sometimes | | 4 | 4 | 6 | 0 | 5 | 0 | |  |
| % rarely/never | | 79 | 94 | 89 | 93 | 94 | 97 | |  |
| How often do you provide smoking cessation counseling (%) | |  |  |  |  |  |  | |  |
| % always/usually | | n/a | 15 | n/a | n/a | 18 | 12 | |  |
| % sometimes | | n/a | 56 | n/a | n/a | 55 | 55 | |  |
| % rarely/never | | n/a | 29 | n/a | n/a | 28 | 33 | |  |
| How often do you ask prescription customers whether they use tobacco? (%) | | |  |  |  |  |  | |  |
| % always/usually | | 19 | 3 | 14 | 7 | 1 | 6 | |  |
| % sometimes | | 25 | 22 | 25 | 29 | 19 | 24 | |  |
| % rarely/never | | 56 | 75 | 61 | 64 | 80 | 70 | |  |
| How often do you ask people whom you counsel for OTC medications whether they use tobacco? (%) | | | |  |  |  |  | |  |
| % always/usually | | 21 | 5 | 11 | 14 | 3 | 12 | |  |
| % sometimes | | 24 | 19 | 19 | 43 | 19 | 12 | |  |
| % rarely/never | | 56 | 76 | 69 | 43 | 79 | 76 | |  |
| How often do you refer patients who use tobacco to the Quitline? (%) | |  |  |  |  |  |  | |  |
| % always/usually | | 22 | 11 | 14 | 21 | 10 | 15 | |  |
| % sometimes | | 29 | 42 | 28 | 36 | 35 | 55 | |  |
| % rarely/never | | 49 | 48 | 58 | 43 | 56 | 30 | |  |
| ^a^ - Differences are statistically significant based on chi-square tests comparing survey versions (p<0.05) | | | |  |  |  |  | |  |
| b - For the Preceptors survey, respondents who indicated they worked in non-community settings or did not interact with patients were not asked this series of questions. | | | | | | | | |  |
