# Supplementary material for: Tobacco sales in pharmacies: a survey of attitudes, knowledge and beliefs of pharmacists employed in student experiential and other worksites in Western New York
Source: BMC Res Notes. 2012 Aug 6;5:413. doi: 10.1186/1756-0500-5-413 (PMC3492148; doi:10.1186/1756-0500-5-413)
Supplement: Additional file 6 — Table 4. Reported barriers to providing tobacco cessation counseling (n=183). [file 1756-0500-5-413-S6.docx]

| Table 4: Reported barriers to providing tobacco cessation counseling (n=183) | | | |  |  |  |
| --- | --- | --- | --- | --- | --- | --- |
|  |  |  | **UB Pharmacy Preceptors (Retail Only)** | | **WNY Pharmacists (Retail Only)** | |
|  | **Total UB Pharmacy Preceptors^b^** | **Total WNY Pharmacists** | **Chain Retailer/Other Retail Setting** | **Independently Owned Pharmacy** | **Chain Retailer/Other Retail Setting** | **Independently Owned Pharmacy** |
|  | (n=66) | (n=117) | (n=40) | (n=15) | (n=81) | (n=35) |
| **Patients' lack of time (%)** |  |  |  |  |  |  |
| Not a barrier | 18 | 12 | 11 | 14 | 13 | 9 |
| Somewhat/occasionally a barrier | 52 | 60 | 53 | 50 | 60 | 64 |
| Definitely/often a barrier | 30 | 28 | 36 | 36 | 28 | 27 |
| **Lack of time/overburdened with other duties (%)** | |  |  |  |  |  |
| Not a barrier | 35 | 23 | 31 | 36 | 23 | 24 |
| Somewhat/occasionally a barrier | 44 | 50 | 44 | 57 | 48 | 58 |
| Definitely/often a barrier | 21 | 27 | 25 | 7 | 30 | 18 |
| **Patients feel it is intrusive (%)** |  |  |  |  |  |  |
| Not a barrier | 42 | 27 | 39 | 15 | 28 | 24 |
| Somewhat/occasionally a barrier | 42 | 53 | 44 | 54 | 52 | 58 |
| Definitely/often a barrier | 17 | 20 | 17 | 31 | 20 | 18 |
| **Pharmacy is not adequately staffed (%)** | |  |  |  |  |  |
| Not a barrier | 47 | 45 | 36 | 71 | 34 | 70 |
| Somewhat/occasionally a barrier | 36 | 40 | 42 | 21 | 46 | 27 |
| Definitely/often a barrier | 17 | 16 | 22 | 7 | 20 | 3 |
| **Lack of support from upper management (%)** | |  |  |  |  |  |
| Not a barrier | 74 | 72 | 58 | 100 | 63 | 91 |
| Somewhat/occasionally a barrier | 14 | 24 | 19 | 0 | 31 | 9 |
| Definitely/often a barrier | 12 | 4 | 22 | 0 | 6 | 0 |
| **Uncomfortable initiating conversation (%)** | |  |  |  |  |  |
| Not a barrier | 64 | 67 | 67 | 29 | 65 | 73 |
| Somewhat/occasionally a barrier | 32 | 28 | 28 | 64 | 29 | 24 |
| Definitely/often a barrier | 5 | 5 | 6 | 7 | 6 | 3 |
| **Don't believe counseling is effective (%)** | |  |  |  |  |  |
| Not a barrier | 79 | 80 | 83 | 64 | 80 | 78 |
| Somewhat/occasionally a barrier | 17 | 17 | 11 | 36 | 16 | 22 |
| Definitely/often a barrier | 5 | 3 | 6 | 0 | 4 | 0 |
| **Lack of training for cessation counseling (%)** | |  |  |  |  |  |
| Not a barrier | 61 | 62 | 61 | 43 | 63 | 59 |
| Somewhat/occasionally a barrier | 36 | 33 | 39 | 43 | 32 | 34 |
| Definitely/often a barrier | 3 | 5 | 0 | 14 | 5 | 6 |
| **Lack of Reimbursement for Smoking Cessation Counseling (%)^b^** | | |  |  |  |  |
| Not a barrier | n/a | 52 | n/a | n/a | 59 | 36 |
| Somewhat/occasionally a barrier | n/a | 33 | n/a | n/a | 28 | 42 |
| Definitely/often a barrier | n/a | 15 | n/a | n/a | 12 | 21 |
| a. Preceptor respondents who indicated they worked in non-community settings or did not interact with patients were not asked this series of questions. | | | | | | |
|  |  |  |  |  |  |  |
| b. This question was included on the WNY Pharmacist survey only. | | | |  |  |  |
