# Supplementary material for: Tobacco sales in pharmacies: a survey of attitudes, knowledge and beliefs of pharmacists employed in student experiential and other worksites in Western New York
Source: BMC Res Notes. 2012 Aug 6;5:413. doi: 10.1186/1756-0500-5-413 (PMC3492148; doi:10.1186/1756-0500-5-413)
Supplement: Additional file 7 — Table 5. Pharmacists’ beliefs about the sale of tobacco products in pharmacies (n=268). [file 1756-0500-5-413-S7.docx]

| Table 5: Pharmacists' beliefs about the sale of tobacco products in pharmacies (n=268) | | | | | |  |  |  |  |
| --- | --- | --- | --- | --- | --- | --- | --- | --- | --- |
|  |  |  |  |  |  |  |  |  |  |
|  |  |  |  | | **UB Pharmacy Preceptors (Retail Only)** | | **WNY Pharmacists (Retail Only)** | |  |
|  |  |  | **Total UB Preceptors** | **Total**  **WNY Pharmacists** | **Chain Retailer/Other Retail Setting** | **Independently Owned Pharmacy** | **Chain Retailer/Other Retail Setting** | **Independently Owned Pharmacy** | **Total for all Respondents** |
|  |  |  | (n=148) | (n=120) | (n=40) | (n=15) | (n=81) | (n=35) | (n=268) |
| **It is inappropriate to sell tobacco products in…** | | | | |  |  |  |  | 77% |
| Community Chain Drug Stores | | |  |  |  |  |  |  |  |
| % agree |  |  | 83 | 70 | 76 | 86 | 67 | 74 |  |
| % disagree | |  | 18 | 30 | 24 | 14 | 33 | 26 |  |
| Community Independent Drug Stores | | | |  |  |  |  |  | 79% |
| % agree |  |  | 85 | 72 | 78 | 86 | 69 | 77 |  |
| % disagree | |  | 15 | 28 | 22 | 14 | 31 | 23 |  |
| Grocery Stores and Wholesale Stores with pharmacies in them | | | | |  |  |  |  | 62% |
| % agree |  |  | 69 | 54 | 65 | 50 | 51 | 57 |  |
| % disagree | |  | 31 | 46 | 35 | 50 | 49 | 43 |  |
| **Prefer to work in a pharmacy that does not sell tobacco products** | | | | |  |  |  |  | 87% |
| % agree  % disagree | | | 90  10 | 83  17 | 89 | 92  8 | 77 | 94 |  |
|  |  |  |  |  | 11 |  | 23 | 6 |  |
| **Support or oppose legislation banning the sale of tobacco in pharmacies?** | | | | |  |  |  |  | 75% |
| % support | |  | 85 | 64 | 92 | 72 | 64 | 62 |  |
| % oppose |  |  | 16 | 12 | 8 | 29 | 10 | 18 |  |
| Not familiar | |  | 0 | 24 | 0 | 0 | 26 | 21 |  |
| ^a^ Underlined values denote statistically significant differences (chi-square test comparing survey versions (p-value<0.05)) | | | | | | | |  |  |
| ^b^ All percentages presented are rounded, and therefore may not total to 100%. | | | | | | |  |  |  |
